# Dietary Patterns and Nutritional Status of Urban School-Children and Adult Females of Reproductive Age in Côte d’Ivoire

**DOI:** 10.1016/j.cdnut.2026.109419

**Published:** 2026-06-30

**Authors:** Katie Ayling, Kim Maasen, Marleen de Jonge, Amoin Georgette Konan, Georges Tiahou, Mory Gbane, Ingeborg Bovee-Oudenhoven, Alida Melse-Boonstra, Elise F Talsma

**Affiliations:** 1 FrieslandCampina., Amersfoort, The Netherlands; 2 Division of Human Nutrition and Health, Wageningen University & Research, Wageningen, The Netherlands; 3 Félix Houphouët-Boigny University, 01 BP V 34 Abidjan 01, Côte d'Ivoire; 4 Ivory Coast Nutrition Society, Commune de Plateau, Boulevard Nanguy Abrogoua, BP V 47 Abidjan, Cote d’Ivoire; 5 Côte d'Ivoire 4Swiss Center for Scientific Research in Côte d'Ivoire (CSRS), Adiopodoumé, Côte d'Ivoire

**Keywords:** Côte d’Ivoire, dietary patterns, schoolchildren, adult females, sociodemographic status, diet quality

## Abstract

**Background:**

Diet strongly influences nutritional status, contributing to the coexistence of undernutrition and excess weight in many low- and middle-income countries. In Côte d’Ivoire, however, dietary patterns and their relationship to nutritional status are not well characterized, limiting evidence-based interventions addressing the dual burden of malnutrition.

**Objectives:**

This study aims to *1*) identify dietary patterns among urban Ivorian schoolchildren and adult females of reproductive age, *2*) assess diet quality by pattern, and *3*) characterize the sociodemographic factors and nutritional status of individuals within each pattern.

**Methods:**

We conducted a cross-sectional dietary survey in 4 cities (Bouaké, Daloa, Abengourou, and Yopougon). Dietary data were collected through one 24-h recall from 443 children (6–12 y) and 441 adult females (15–49 y); a second recall was administered to 12% of participants. Dietary patterns were derived using latent class analysis. Associations with nutritional status, sociodemographic indicators, diet quality, diet diversity, and nutrient adequacy (Probability of Adequacy) were examined using the 3-step bias-adjusted approach.

**Results:**

Four dietary patterns were identified for each group. Two were found in both children and adult females (“Fish, oil, and vegetables” and “Dairy and sweet”), whereas children had “Poultry and fruit” and “Refined, red meat, and vegetable” patterns, and adult females had “Poultry, red meat, and fruit” and “Low energy intake” patterns. The fish and dairy patterns, more common in Bouaké and Daloa, were associated with lower education levels (and with occupation, housing, and dependent children among adult females only), yet showed higher diet quality, characterized by greater dietary diversity and nutrient adequacy. In contrast, the poultry (children) and low-intake (adult females) patterns showed poorer dietary diversity and nutrient adequacy.

**Conclusions:**

Dietary patterns among urban Ivorian children and adult females were largely comparable. Promoting familiar, fish- and vegetable-based diets may enhance nutrient adequacy. The early stage of nutrition transition suggests a window of opportunity for preventive policy action.

This trial was registered at clinicaltrials.gov as NCT06175130.

## Introduction

Côte d’Ivoire, like many low- and middle-income countries (LMICs), is now experiencing a “triple burden” of malnutrition, characterized by the coexistence of undernutrition and micronutrient deficiencies and rising rates of overweight and obesity [1,2]. This burden is not uniform, varying across regions, age groups, and genders. More than half (51%) of adult females are overweight or obese, whereas adult females and children both continue to experience multiple micronutrient inadequacies [3]. Among urban school-aged children (6–12 y), most (82%) have a healthy weight, yet recent evidence indicates increasing overweight in this group [4]. These differing trends between adult females and children reflect broader regional shifts in sub-Saharan Africa, where rapid urbanization and dietary changes are driving complex and overlapping forms of malnutrition [[5], [6], [7], [8]].

The high prevalence of overweight and obesity observed among adult females in Côte d’Ivoire likely reflects broader dietary changes accompanying economic and social transformation. Across sub-Saharan Africa, these shifts are evident as monotonous cereal-based diets give way to more diverse patterns that include animal-sourced foods, fruits and vegetables, and processed or out-of-home foods [9,10]. Such changes typically accompany economic growth and urbanization and are concurrent with lower physical activity [11]. Importantly, they do not affect everyone equally, with greater differentiation by socioeconomic status than urban–rural residence [9]. Despite these regional trends, relatively little is known about Côte d’Ivoire, which has a distinct nutritional and food system profile from other West African countries. Few studies have characterized dietary behaviors across population groups to identify shared and group-specific drivers of malnutrition within the same socioeconomic and food system context. Understanding how these dietary shifts manifest within and between groups is essential for addressing emerging nutritional inequalities [[12], [13], [14]].

Dietary pattern analysis offers a useful approach for addressing this evidence gap. By examining combinations of foods rather than individual nutrients, this approach provides insight into how overall diets relate to sociodemographic factors, urbanization, and exposure to processed, energy-dense foods [15,16]. In Côte d’Ivoire, where adult females and children face distinct but overlapping malnutrition challenges, understanding dietary patterns may clarify how urbanization and changing dietary behaviors contribute to both undernutrition and excess weight. In this study, we aimed to *1*) identify dietary patterns of urban Ivorian school-aged children and adult females, *2*) examine the similarities and differences between these patterns, *3*) assess diet quality by pattern, and *4*) characterize the sociodemographic factors and nutritional status of individuals within each pattern.

## Methods

### Study design and participants

We conducted a cross-sectional study in February 2021 to assess dietary intake among Ivorian school-aged children and adult females of reproductive age. Data were collected from 443 school-aged children (6–12 y) and 441 adult females of reproductive age (15–49 y) in the following 4 cities of Côte d’Ivoire: Abidjan (Yopougon), the largest and most urbanized city (5.5 million inhabitants); Bouaké, the second largest city (930k); Daloa, a mid-sized commercial city (300,000); and Abengourou, a smaller regional capital (100,000). The study received ethical approval from the National Ethics and Research Committee of Côte d’Ivoire. Informed consent was obtained from all adult female participants and from a parent or caregiver for each child.

### Sampling

Participants (children and adult females separately) were selected using a 2-stage random sampling procedure. First, neighborhoods were selected by simple random sampling from a list of neighborhoods in each city, according to the General Population and Housing Census (Recensement Général de la Population et de l’Habitat) 2014. The number of selected neighborhoods per city was determined using probability proportional to city size. Within each neighborhood, households were selected by using the random walk method. Eligible participants were assessed as apparently healthy by a physician at the time of the study and were either children (6–12 y) or adult females (15–49 y). Only 1 participant was selected per household; hence, adult females and children did not reside in the same households. In households where >1 eligible individual was present, selection was performed using the Kish table method.

### Data collection

Trained field investigators conducted face-to-face interviews with all participants (for children, a caregiver was also present). Data were collected using an electronic survey tool KoboCollect (version 1.25.1, KoboToolbox). Height and weight were measured using a standardized height board and scale. Demographic and socioeconomic characteristics, including caregiver’s educational level and occupation, housing type, number of people living in the household, and daily household expenditure on food were collected by questionnaires. Dietary intake was using an interviewer-administered multiple pass 24-h recall [17]. Portion sizes of foods and beverages were estimated based on a photographic food atlas [18]. A second 24-h recall was administered for 12% of the participants on nonconsecutive days with ≥7 d and at most 18 d between the 2 recalls. Further details on dietary intake, sociodemographic measures, anthropometry, and data cleaning are reported elsewhere [3].

### Statistical analysis

All analyses were conducted in RStudio (version 4.3.1, R statistical software) and Latent Gold (version 6.1) [19]. Descriptive statistics were reported as mean ± SD for continuous variables and frequencies (%) for categorical variables.

### Characterization of dietary patterns in children and adult females

Dietary patterns were identified using latent class analysis (LCA). Of the 25 food groups in the Global Diet Quality Score (GDQS) classification described below, 9 with <10 consumers were excluded, leaving 16 food groups for the LCA [20]. Total daily intake (g/d) was calculated per food group and categorized into ordinal variables (nonconsumption, low, or high intake) based on median intake among consumers, following the studies by Dalmartello et al. [21,22]: values are provided in Supplemental Tables 1 (children) and 2 (adult females).

Models with 1 to 5 classes were estimated. The optimal number of patterns was selected based on model fit, considering lower values of Bayesian information criterion (BIC) and Akaike information criterion, parsimony, and model interpretability. Additional diagnostic indices, such as entropy *R*^2^, were also examined [23].

The LCA assumption of local independence between food groups (i.e., food groups are statistically independent of one another) was checked by examining bivariate residuals (BVRs). BVRs standardized measures of the residual association between pairs of food groups after accounting for class membership. Where BVR >4, residual correlations were permitted when theoretically justified (e.g., nuts and seeds and other vegetables, reflecting common co-preparation in Ivorian dishes).

Total energy intake (kilocalorie) was included as a covariate, both as a predictor of class membership and as a direct effect on all food group indicators, to ensure that dietary patterns reflected composition rather than total energy intake [24]. Pattern-specific mean intakes were estimated using a bias-adjusted 3-step approach, with food group intakes treated as distal outcomes and model parameters estimated using maximum likelihood (ML) with robust SEs and class-dependent variances [25]. SDs of the means were derived from the SEs.

### Bivariate associations between dietary patterns and participant/household characteristics

Bivariate associations between dietary patterns and participant/household characteristics were examined within the LCA framework using Wald tests, with *P* ≤ 0.05 considered statistically significant. Continuous variables were examined using the Bolck–Croon–Hagenaars method and categorical variables using ML estimation, both with robust SEs [26]. When overall Wald tests indicated significant differences across dietary patterns, pairwise comparisons were performed, with *P* ≤ 0.05 considered statistically significant.

### Sociodemographic status

Socioeconomic status (SES) was derived using principal component analysis (PCA) in R Studio (packages: “Corr” and “ggcorrplot”), based on the following 4 commonly used indicators: housing type (apartment, villa/house, and common commune/shack), household food expenditure (continuous, per day), education (none, primary, secondary, and tertiary), and occupation (formal employment, informal employment, and unemployed/student). However, no component with an eigenvalue ≥1 was identified. Consequently, a composite SES score was not constructed, and the individual SES indicators were analyzed separately.

### Nutritional status

Nutritional status was assessed using anthropometric measures, compared with age- and sex-specific WHO standards, and calculated using WHO AnthroPlus Software (version 1.0.4) [27]. For children (5–19 y), obesity was defined as BMI-for-age >+2 SD, overweight as >+1 SD, thinness as <−2 SD and stunting as height-for-age <−2 SD [28]. For adults, BMI was calculated as weight in kilograms divided by height in meters squared (kg/m^2^) and categorized as underweight (<18.5), normal (18.5–24.9), overweight (25–29.9), and obese (≥30) [29].

### Diet quality

Diet quality was evaluated using the following 3 indicators: *1*) the GDQS [20], *2*) the Diet Diversity Score (DDS) [30], and *3*) nutrient adequacy.

#### GDQS

Individual foods based on the first-day 24-h recall were classified into the corresponding GDQS food groups and reviewed by 4 experts (KA, KM, EFT, and AM-B). The GDQS is a validated, food-based measure of diet quality that reflects risk of nutrient inadequacy and diet-related noncommunicable disease (NCD) [31,32]. It includes 25 food groups: 16 considered healthy (GDQS+) and 9 associated with higher disease risk (GDQS−; 7 unhealthy and 2 unhealthy at excessive intakes), with total scores ranging from 0 to 49 and higher scores reflecting healthier diets [31,32]. Population-level GDQS categories were defined as ≥23 points (low risk of NCDs and nutrient inadequacy); 15 to 23 points (moderate risk), and <15 points (high risk). We reported the mean GDQS and the prevalence of participants in each risk category for both groups [10].

#### DDS

Diet diversity was assessed using the DDS, which reflects the number of different food groups consumed over a 24-h period and serves as an indicator of dietary variety and micronutrient adequacy. Individual foods from the first-day 24-h dietary recall were classified into 10 food groups following the DDS framework [30]. Participants received 1 point per food group consumed (>15 g), summed to a total DDS (range 0–10). For adult females, we also calculated Minimum Dietary Diversity for Women, which uses the same 10 food groups but applies a validated cutoff of ≥5 food groups, representing the minimum dietary diversity associated with a higher likelihood of adequate micronutrient intake at a population level [33]. For children, we reported the mean DDS and the proportion consuming ≥5 food groups for a descriptive comparison, while acknowledging that this cutoff has not been validated for children [30].

#### Nutrient adequacy

Usual intakes of 11 micronutrients (calcium, iron, zinc, vitamin A, thiamin, riboflavin, niacin, vitamin B-6, folate, vitamin B-12, and vitamin C) were estimated using the Statistical Program to Assess Dietary Exposure (SPADE; version 4.1.1, RIVM) in R Studio (Packages: SPADE.RIVM), adjusted for within-person variation using repeat 24-h dietary recalls available for 12% of participants. Nutrient adequacy was then assessed using the estimated average requirement (EAR) cut-point method against age- and sex-specific EARs, selected for their public health relevance in the West African region [34,35]. Harmonized average requirement from Allen et al. [25] were used, as Côte d’Ivoire lacks national recommendations. Iron adequacy among adult females was assessed using the WHO full requirement distribution given the skewed nature of iron requirements [30], with moderate levels of bioavailability assumed for iron (10%) and zinc (25%) [36], consistent with Côte d’Ivoire’s Human Development Index classification [37].

Pattern-specific usual intakes were estimated using SPADE’s subgroup functionality [50 bootstrap iterations; 95% confidence interval (CI)]. Differences between patterns were assessed by non-overlap of 95% CIs.

### Sensitivity analysis

Following 3 sensitivity analyses were conducted: *1*) comparison of model solutions with and without energy as covariate, *2*) a combined population model including both groups, and *3*) evaluation of relaxing local independence assumptions.

## Results

### Population characteristics

Participants were sampled across Abidjan (36%), Bouaké (29%), Daloa (26%), and Abengourou (9%). Among school-aged children, 10 records were judged implausible and excluded, leaving 434 school-aged children (mean age: 9.1 ± 1.9 y; 55% female), of whom 54 completed a second recall. In this group, 82% had a healthy BMI-for-age, 7% had overweight/obesity, and 11% had thin or underweight. Additionally, 3% of children had stunting, reflecting chronic undernutrition. Among adult females, 7 records were excluded as implausible, leaving 434 (mean age: 29.4 ± 8.8 y), of whom 52 completed a second recall. In this group, 44% had a normal weight, 51% had overweight or obese, and 7% had thinness or underweight.

### Identification of dietary patterns

For both children and adult females, a 4-cluster (dietary pattern) solution was selected based on a balance between statistical fit and interpretability (Supplemental Table 3). Although the 2-cluster models had the lowest BIC, the 4-cluster models showed a more acceptable entropy, lower BVRs, and yielded interpretable solutions.

### Characteristics of dietary patterns and diet quality

**FIGURE 1:**
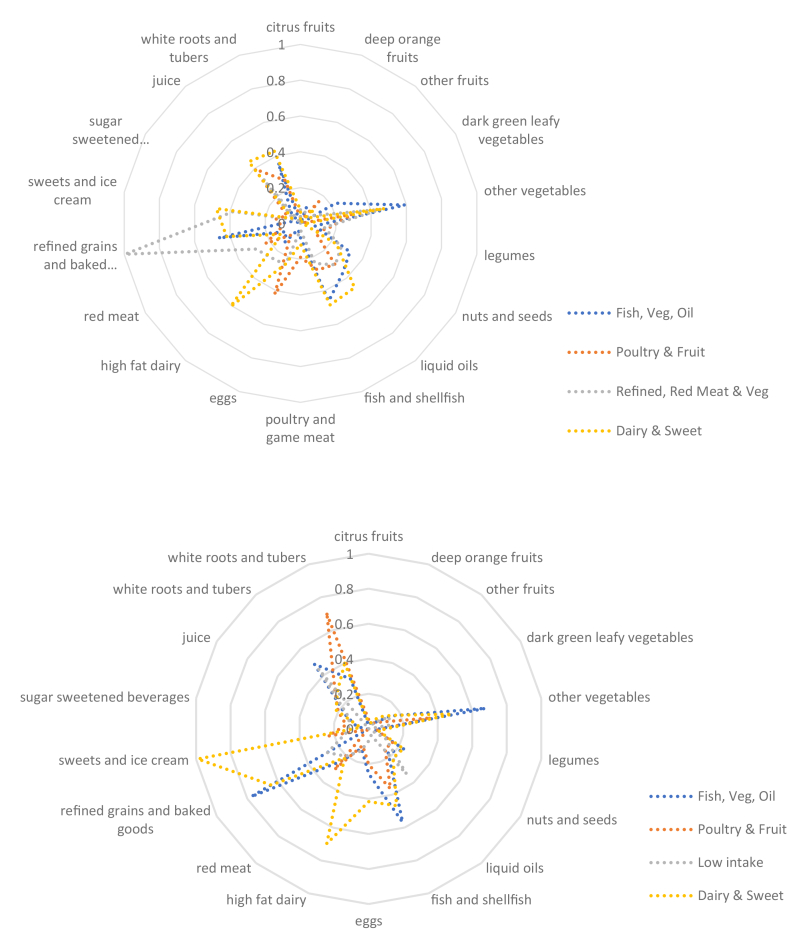
Dietary pattern classification based on the proportion of consumers in the highest intake category for each food group among children (top) and adult females (bottom). The figure highlights the food groups most frequently consumed at high levels within each dietary pattern. Classification into intake categories and the definition of the proportion of consumers are described in the Methods section. The corresponding proportions for all intake categories, including nonconsumers, are reported in Supplemental Tables 1 and 2 for children and adult females, respectively.

Among children, 4 distinct dietary patterns were identified and named (Figure 1, Table 1). The most common pattern, named “Dairy and sweet” (36% of children), was characterized by higher consumption of dairy, fruits other than vitamin A-rich or citrus varieties, juice, and sweet foods than the other patterns. This pattern showed intermediate diet healthiness (GDQS), the highest diversity (DDS), and the highest micronutrient adequacy than the other patterns. The second largest, “Fish, vegetables, and oil” pattern (25%), included higher consumption of dark green leafy vegetables, oils, fish, deep orange fruits, and white roots and tubers. It showed the highest total healthiness (GDQS), the lowest diversity (DDS), and intermediate micronutrient adequacy than the other patterns. The “Poultry, eggs, fruits” pattern (23%) showed the lowest total GDQS, DDS, and nutrient adequacy, reflecting low consumption of healthy foods (GDQS+) and high consumption of unhealthy foods (GDQS−). The smallest, “Refined, red meat, and vegetables” pattern (16%), included higher consumption of red meat, refined grains, legumes, nuts and seeds, and vegetables, and showed intermediate GDQS, DDS, and nutrient inadequacy.

**TABLE 1:** Food group intake (g/d) and diet quality indicators by dietary patterns urban Ivorian school-aged children (6–12 y), 2021

	“Dairy and sweet”	“Fish, vegetable, and oil”	“Poultry and fruit”	“Refined, red meat, and vegetable”	Total	Wald (*P* value)
Proportion, *n* (%)	156 (25)	108 (24)	100 (21)	71 (16)	435	—
Total energy (kcal/d)	2325 ± 74	2156 ± 93	1615 ± 81	2410 ± 129	2134 ± 38	50 (<0.001)
Healthy food group intake^1^ (g/d)						
Citrus fruits^2^ (g/d)	0 ± 0	0 ± 0	142 ± 232^5^	0 ± 0	32.6 ± 111	38 (<0.001)
Deep orange fruits^2^ (g/d)	0 ± 0	167 ± 324^5^	0 ± 0	0 ± 0	41.3 ± 161	29 (<0.001)
Other fruits^2^ (g/d)	283 ± 1193^5^	0 ± 0	0 ± 0	0 ± 0	101 ± 715	9 (0.0031)
Dark green leafy vegetables^2^ (g/d)	0 ± 0	50.1 ± 70.9^5^	0 ± 0	0 ± 0	12.4 ± 35.2	54 (<0.001)
Cruciferous vegetables^2^ (g/d)	55.4 ± 415	0 ± 0	0 ± 0	0 ± 0	19.9 ± 249	3 (0.0950)
Deep orange vegetables^2^ (g/d)	0 ± 0	0 ± 0	38.6 ± 193	0 ± 0	8.9 ± 92.3	4 (0.0450)
Other vegetables^2^ (g/d)	66 ± 154	116 ± 138	29.2 ± 27.2	258 ± 307^5^	101 ± 145	150 (<0.001)
Legumes^2^ (g/d)	0 ± 0	0 ± 0	76.8 ± 132	235 ± 331^5^	56.1 ± 147	72 (<0.001)
Deep orange tubers^2^ (g/d)	0 ± 0	0 ± 0	0 ± 0	0 ± 0	0 ± 0	—
Nuts and seeds^2^ (g/d)	0 ± 0.1	53.6 ± 72.9	0 ± 0.1	184 ± 249^5^	43.4 ± 112	87 (<0.001)
Whole grains^2^ (g/d)	11.8 ± 49.1	0 ± 0	0 ± 0	0 ± 0	4.2 ± 29.4	9 (0.0028)
Liquid oils^2^ (g/d)	120 ± 109	240 ± 224^5^	20.2 ± 45.3	3.3 ± 9	108 ± 132	302 (<0.001)
Fish and shellfish^2^ (g/d)	58.4 ± 50.9	120 ± 118^5^	19.7 ± 59.2	17.9 ± 32.7	58.2 ± 75.4	129 (<0.001)
Poultry and game meat^2^ (g/d)	0 ± 0	0 ± 0	142 ± 144^5^	0 ± 0	32.5 ± 68.8	97 (<0.001)
Low fat dairy^2^ (g/d)	0 ± 0	0 ± 0	0 ± 0	0 ± 0	0 ± 0	—
Eggs^2^ (g/d)	33 ± 79.9	0 ± 0	55 ± 80.7^5^	51.9 ± 143^5^	33 ± 58.5	177 (<0.001)
High-fat dairy^2^^,^^3^ (g/d)	97.2 ± 217^5^	0 ± 0.2	0.1 ± 1	20.9 ± 70.1	38.4 ± 143	31 (<0.001)
Red meat^2^^,^^3^ (g/d)	22 ± 17.7	0 ± 0.1	43 ± 86.3	80.3 ± 98.3^5^	30.9 ± 51.7	309 (<0.001)
Unhealthy food groups intake^1^ (g/d)						
Processed meat^2^ (g/d)	2.7 ± 40.8	0 ± −4.5	0 ± −4.3	0 ± −3.6	1 ± 24.5	1 (0.4100)
Refined grains and baked goods^2^ (g/d)	446 ± 296	493 ± 285	235 ± 160	997 ± 380^5^	500 ± 276	288 (<0.001)
Sweets and ice cream^2^ (g/d)	56.2 ± 106^5^	0.6 ± 1.5	3.7 ± 11.2	14.3 ± 12.7	23.5 ± 66.6	102 (<0.001)
Sugar-sweetened beverages	194 ± 480	0 ± 0	0 ± 0	0 ± 0	69.5 ± 288	25 (<0.001)
Juice^2^ (g/d)	236 ± 245^5^	0 ± 0	120 ± 120	0 ± 0	112 ± 159	238 (<0.001)
White roots and tubers^2^ (g/d)	370 ± 423	452 ± 528^5^	211 ± 159	1 ± 5	293 ± 236	689 (<0.001)
Purchased deep-fried foods	0 ± 0	0 ± 0	0 ± 0	0 ± 0	0 ± 0	—
Diet quality						
GDQS total^2^ (range: 0–49)	15.1 ± 3.2^a^	17.2 ± 3.4^5^^,b^	14.4 ± 3.4^a^	15.6 ± 3.8^a^	15.6 ± 2.9	36 (<0.001)
GDQS plus^2^ (range: 0–32)	5.8 ± 2.8^a^	6.5 ± 3.3^5^^,b,d^	4.7 ± 3^c,e^	4.9 ± 4^a,d,e^	5.6 ± 2.7	18 (<0.001)
GDQS minus^2^ (range: 0–17)	9.2 ± 2^5^^,a^	10.7 ± 1.2^b^	9.7 ± 2^c^	10.8 ± 2^b^	10 ± 1.6	55 (<0.001)
GDQS category, high risk^4^ (%)	43 (35–52)^a^	26 (16–25)^b^	60 (49–71)^5^^,a^	45 (32–73)^c^	43 (38–48)	17 (0.0073)
GDQS category, moderate risk (%)	56 (48–65)	73 (63–83)	40 (29–51)	48 (35–53)	55 (51–60)	
GDQS category, low risk^4^ (%)	0 (−0.5 to 0.6)	1 (−1.1 to 4)	0 (0–0.1)	6 (0.2–6)^5^	1 (0.3–2)	
DDS score^2^	4.2 ± 1.1^5^^,a^	3.5 ± 1.2^b^	3.5 ± 1.2^c^	3.9 ± 1.4^a,b,c^	3.8 ± 1	27 (<0.001)
DDS, adequate diversity,^4^ (%) (% ≥5 food groups)	37 (28–45)^5^^,a^	14 (6–21)^b^	12 (4–23)^b^	20 (1–31)^a,b^	23 (27–19)	16 (<0.001)
Calcium inadequacy^4^ (%)	90 (83–97)^5^	95 (85–100)	99 (97–100)	97 (91–100)	93 (91–95)	—
Riboflavin inadequacy^4^ (%)	50 (38–80)	66 (56–76)	71.5 (57–86)	47 (29–64)^5^	59 (53–65)	
Folate inadequacy^4^ (%)	29 (42–38)^5^	34 (21–44)	47 (33–72)	43 (22–61)	36 (33–38)	
Zinc inadequacy^4^ (%)	48 (41–56)	56 (45–71)	74 (66–86)	36 (22–51)^5^	54 (51–57)	
Vitamin A inadequacy^4^ (%)	46 (36–57)^5^	55 (42–70)	64 (51–74)	61 (43–79)	54 (50–57)	
Vitamin C inadequacy^4^ (%)	44 (29–100)	41 (32–54)^5^	51 (37–69)	43 (30–60)	43 (38–54)	
Thiamin inadequacy^4^ (%)	9.9 (3.9–17)^5^	18 (8.5–28)	25 (11–36)	17 (9.5–28)	18 (11–33)	
Vitamin B-6 inadequacy^4^ (%)	26 (18–36)^5^	41 (22–58)	42 (32–55)	30 (15–41)	34 (30–36)	
Iron inadequacy^4^ (%)	17 (9.8–25)^5^	28 (16–38)	39 (24–60)	21 (4.9–55)	25 (20–29)	
Niacin inadequacy^4^ (%)	5.3 (2.3–9.3)^5^	11 (4–18)	23 (13–35)	8.2 (2.9–14)	12 (7.9–20)	
Vitamin B-12 inadequacy^4^ (%)	2.1 (0.1–5.6)^5^	6.5 (1.4–12)	15 (4.5–29)	18 (1.7–36)	8 (5.9–13)	

*P* values are reported in Supplemental Table 4.

Abbreviations: DDS, Diet Diversity Score; GDQS, Global Diet Quality Score.

^a,b,c^If the mean of a variable has the same superscript (e.g., a and a) for 2 dietary patterns, the pairwise comparison between the 2 dietary patterns had *P* ≥ 0.05; if the means have a different superscript (e.g., a and b), the pairwise comparison between the 2 dietary patterns had a *P* < 0.05.

1 Healthy and unhealthy food group intake were classified according to the GDQS food group definitions [20)].

2 Values are mean g/d ± SD; zero values reflect estimated class means from the Bolck–Croon–Hagenaars method and indicate that the food group is not characteristic of that dietary pattern; they do not imply complete absence of consumption.

3 Food groups considered unhealthy when consumed in excessive amounts according to the GDQS.

4 Values are % (95% confidence interval).

5 Indicates food groups that contributed most to the characterization of each dietary pattern.

Among adult females, 4 corresponding patterns were identified (Table 2). The largest, the “Fish, vegetables, and oils” pattern (32%), was characterized by higher intakes of deep orange fruits, vegetables, fish, oils, and refined grains and showed high GDQS, intermediate DDS, and intermediate micronutrient adequacy than the other patterns. The “Poultry, red meat, and fruit” pattern (26%) included higher intake of poultry, citrus fruit, red meat, white roots and tubers, and intermediate GDQS, DDS, and nutrient adequacy. The “Dairy and sweet” pattern (24% of adult females) was characterized by higher intakes of fruits, legumes, eggs, high-fat dairy, sweet foods, and juice and showed the highest GDQS, DDS, and nutrient adequacies than the other patterns. Finally, the “Low energy intakes” pattern (18%) reflected generally lower intake across all food groups and the lowest mean energy intake (1242 kcal vs. mean of 2720 kcal in the overall population), the lowest GDQS, DDS, and nutrient adequacy than the other patterns.

**TABLE 2:** Food group intake (g/d) and diet quality indicators by dietary patterns among Ivorian female adults (15–49-y old), 2021

	“Dairy and sweet”	“Fish, vegetable, and oil”	“Poultry, red meat, and fruit”	“Low energy intake”	Total	Wald (*P* value)
Proportion, *n* (%)	104 (22)	139 (32)	112 (26)	79 (18)	434	
Total energy (kcal/d)	3170 ± 1920	3219 ± 1834	2727 ± 1176	1242 ± 485	2720 ± 1312	50 (<0.001)
Healthy food group intake^1^ (g/d)						
Citrus fruits^2^ (g/d)	0 ± 0	0 ± 0	143 ± 343^5^	0 ± 0	37.1 ± 175	20 (<0.001)
Deep orange fruits^2^ (g/d)	0 ± 0	208 ± 747^5^	0 ± 0	0 ± 0	66.4 ± 422	11 (0.00)
Other fruits^2^ (g/d)	106 ± 374^5^	0 ± 0	0 ± 0	0 ± 0.4	25.5 ± 183	9 (0.04)
Dark green leafy vegetables	24.2 ± 63.7	58.7 ± 113^5^	0 ± 0	0 ± 0	24.5 ± 70.4	54 (<0.001)
Cruciferous vegetables^2^ (g/d)	0 ± 0	0 ± 0	0 ± 0	73.8 ± 137^5^	13.4 ± 58.6	23 (<0.001)
Deep orange vegetables^2^ (g/d)	0 ± 0	0 ± 0	61.8 ± 512	0 ± 0	16 ± 261	2 (0.20)
Other vegetables^2^ (g/d)	146 ± 249	241 ± 326^5^	72 ± 80.5	28.4 ± 32.4	136 ± 200	121 (<0.001)
Legumes^2^ (g/d)	132 ± 550^5^	0 ± 0	0 ± 0	0 ± 0	31.7 ± 269	6 (0.01)
Deep orange tubers^2^ (g/d)	0 ± 0	0 ± 0	0 ± 0	0 ± 0	0 ± 0	—
Nuts and seeds^2^ (g/d)	108 ± 255^5^	84.9 ± 157	0 ± 0.1	0 ± 0.2	53.1 ± 137	76 (<0.001)
Whole grains^2^ (g/d)	0 ± 0	0 ± 0	19.9 ± 215	0 ± 0	5.1 ± 110	1 (0.33)
Liquid oils^2^ (g/d)	151 ± 185	254 ± 297^5^	45.1 ± 79.6	6.8 ± 15.3	130 ± 196	175 (<0.001)
Fish and shellfish^2^ (g/d)	122 ± 254^5^	159 ± 206^5^	59 ± 90.1	19.5 ± 26.4	98.7 ± 111	196 (<0.001)
Poultry and game meat^2^ (g/d)	0 ± 0	0 ± 0	130 ± 152^5^	0 ± 0	33.7 ± 77.5	82 (<0.001)
Low fat dairy^2^ (g/d)	0 ± 0	0 ± 0	0 ± 0	0 ± 0	0 ± 0	—
Eggs^2^ (g/d)	89.7 ± 115^5^	60.6 ± 76.5	0 ± 0	0 ± 0	40.9 ± 69.5	157 (<0.001)
High-fat dairy^2^^,^^3^ (g/d)	93.5 ± 144^5^	0 ± 0.4	0 ± 0.2	13.1 ± 23	24.8 ± 73	60 (<0.001)
Red meat^2^^,^^3^ (g/d)	24.1 ± 42.3	0 ± 0.1	103 ± 101^5^	45.4 ± 33.6	40.8 ± 58.6	294 (<0.001)
Unhealthy food groups intake^1^ (g/d)						
Processed meat^2^ (g/d)	88.6 ± 920	0 ± 0	0 ± 0	0 ± 0	21.2 ± 450	1 (0.33)
Refined grains and baked goods	857 ± 598^5^	935 ± 500^5^	207 ± 203	396 ± 310	630 ± 440	332 (<0.001)
Sweets and ice cream^2^ (g/d)	68.3 ± 126^5^	0 ± 0.1	2.7 ± 9.5	0.8 ± 3.7	17.2 ± 63.3	37 (<0.001)
Sugar-sweetened beverages	0 ± 0	0 ± 0	327 ± 512	0 ± 0	84.5 ± 261	46 (<0.001)
Juice^2^ (g/d)	219 ± 560^5^	0 ± 0	201 ± 314	0 ± 0	104 ± 268	89 (<0.001)
White roots and tubers^2^ (g/d)	467 ± 675	300 ± 286	759 ± 445^5^	84.2 ± 282	420 ± 375	209 (<0.001)
Purchased deep-fried foods	0 ± 0	0 ± 0	0 ± 0	0 ± 0	0 ± 0	—
Diet quality						
GDQS total^2^ (range: 0–49)	16.7 ± 3.1^5^^,a^	16.5 ± 3^5^^,a^	15.3 ± 2.9^b^	13.8 ± 2.5^c^	15.8 ± 2.7	36 (<0.001)
GDQS plus^2^ (range: 0–32)	6.4 ± 2.9^a^	6.2 ± 2.7^a^	5.1 ± 2.7^b^	2.4 ± 1.5^c^	5.3 ± 2.3	18 (<0.001)
GDQS minus^2^ (range: 0–17)	10.3 ± 2^b^	10.3 ± 1.5^a^	10.1 ± 1.8^a^	11.4 ± 1.9^c^	10.5 ± 1.6	55 (<0.001)
GDQS category, high risk^4^ (%)	27 (18–35)^a^	32 (21–39)^a^	43 (33–53)^a^	68 (56–38)^5^^,b^	40 (25–45)	18 (<0.001)
GDQS category, moderate risk^4^ (%)	72 (62–81)	66 (57–75)	56 (46–66)	32 (20–83)	59 (54–63)	
GDQS category, low risk^4^ (%)	2 (−0.8 to 5)^5^	2 (−0.3 to 5)^5^	0.6 (−1.2 to 3)	0 (0–2)	1 (0.3–2)	
DDS score^2^	4.6 ± 0.94^5^^,c^	3.8 ± 0.92^a^	3.4 ± 0.87^b^	2.7 ± 0.69^d^	3.7 ± 0.85	27 (<0.001)
DDS, adequate diversity^4^ (%) (% ≥5 food groups)	52 (42–62)^c^	19 (9–26)	6 (1–9)^5^^,a^	1 (1–3)^5^^,a^	20 (22–17)	16 (<0.001)
Calcium inadequacy^4^ (%)	81 (64–95)^5^	94 (89–97)	95 (90–98)	99 (98–100)	91 (89–93)	—
Riboflavin inadequacy^4^ (%)	75 (45–100)^5^	90 (72–100)	87 (69–100)	99 (94–100)	87 (85–88)	
Folate inadequacy^4^ (%)	47 (31–65)	57 (41–71)	44 (33–63)^5^	94 (88–100)	58 (55–61)	
Zinc inadequacy^4^ (%)	44 (29–58)^5^	51 (35–61)	54 (412–79)	90 (85–97)	56 (53–59)	
Vitamin A inadequacy^4^ (%)	42 (32–55)^5^	50 (37–60)	66 (49–82)	78 (59–97)	56 (52–60)	
Vitamin C inadequacy^4^ (%)	38 (14–56)^5^	41 (27–53)	40 (27–56)	86 (73–100)	48 (44–51)	
Thiamin inadequacy^4^ (%)	34 (18–49)	37 (27–45)	19 (0–34)^5^	96 (93–100)	44 (40–47)	
Vitamin B-6 inadequacy^4^ (%)	16 (0.7–27)^5^	22 (15–31)	25 (16–25)	83 (71–94)	30 (42–35)	
Iron inadequacy^4^ (%)	35 (20–47)	39 (29–48)	29 (24–35)	93 (84–97)^5^	45 (37–52)	
Niacin inadequacy^4^ (%)	17 (7.9–26)	12 (4.1–21)^5^	16 (7.3–25)	71 (54–89)	24 (20–27)	
Vitamin B-12 inadequacy^4^ (%)	8 (0.3–20)^5^	8.7 (4.7–13)	13.8 (2.9–96)	52 (40–65)	16 (11–18)	

*P* values are reported in Supplemental Table 4.

Abbreviations: DDS; Diet Diversity Score; GDQS; Global Diet Quality Score.

^a,b,c^If the mean of a variable has the same superscript (e.g., a and a) for 2 dietary patterns, the pairwise comparison between the 2 dietary patterns had *P* ≥ 0.05; if the means have a different superscript (e.g., a and b), the pairwise comparison between the 2 dietary patterns had a *P* < 0.05.

1 Healthy and unhealthy food group intake were classified according to the GDQS food group definitions [20].

2 Values are mean g/d ± SD; zero values reflect estimated class means from the Bolck–Croon–Hagenaars method and indicate that the food group is not characteristic of that dietary pattern; they do not imply complete absence of consumption.

3 Food groups considered unhealthy when consumed in excessive amounts according to the GDQS.

4 Values are % (95% confidence interval).

5 Indicates food groups that contributed most to the characterization of each dietary pattern.

### Associations between dietary patterns and sociodemographic factors

Dietary patterns were associated with city of residence and maternal education among children (Table 3), and with city of residence and education among adult females (Table 4). In adult females, additional associations were observed with ethnicity, occupation, housing type, and number of dependent children (Wald test ≤ 0.05).

**TABLE 3:** General characteristics of the urban Ivorian school-aged children (6–12-y old) population by dietary pattern, 2021

	“Dairy and sweet”	“Fish, vegetable, and oil”	“Poultry and fruit”	“Refined, red meat, and vegetable”	Total	Wald (*P* value)
*n* (%)	156 (25)	108 (24)	100 (21)	71 (16)	434	
kcal/d ± SD	2325 ± 74	2156 ± 93	1615 ± 81^2^	2410 ± 129	2134 ± 38	50 (<0.001)^3^
Participant						
Age (y)	9.1 ± 2.1^a^	9 ± 2.2^a^	9.1 ± 2.2^a^	9.2 ± 2.2^a^	9.1 ± 1.9	0 (0.94)
Mother’s age (y)	35.8 ± 9.8^a^	36.3 ± 10.7^a^	35.5 ± 8.6^a^	36.9 ± 10.2^a^	36 ± 8.6	1 (0.82)
Sex (%)						
Female	55 (46–64)	51 (40–63)	63 (52–74)	50 (37–64)	55 (51–60)	3 (0.440)
City (%)						
Abengourou	6 (2–14)^a^	10 (3–16)^a^	10 (4–15)^b^	11 (3–42)^a,b^	9 (6–14)	20 (0.017)^3^
Abidjan (Youpougon)	33 (22–40)	19 (13–29)	53^2^ (42–64)	40 (26–53)	35 (31–39)	
Bouke	31^2^ (21–38)	42^2^ (31–53)	20 (13–29)	20 (12–31)	29 (24–34)	
Daloa	30^2^ (20–37)	29 (19–38)	18 (12–27)	29 (17–40)	27 (21–31)	
Ethnicity (%)						
Akan	37 (28–46)^a^	46 (35–57)^b^	50 (39–62)^a^	34 (23–47)^a^	42 (36–46)	22 (0.250)
Gour	8 (3–10)	9 (2–15)^2^	5 (−0.2 to 10)	3 (−2.5 to 8)	7 (4–12)	
Krou	18 (14–24)	0.4 (−0.8 to 2)	24 (15–34)^2^	31 (19–43)	17 (11–42)	
Mande du Nord	21 (11–28)	29 (19–37)	11 (3–18)	16 (7–26)^2^	20 (16–21)	
Mande du Sud	7 (3–9)	6 (0.8–10)^2^	5 (0–12)	9 (2–16)	6 (4–12)	
Non-Ivorian	5 (1–12)^2^	4 (−0.1 to 9)	1 (−1.8 to 4)	6 (−0.5 to 13)	4 (2–6)	
Other	4 (0.3–8)^2^	6 (0.9–12)	4 (−0.2 to 8)	1 (−1.9 to 4)	4 (2–6)	
Weight (kg)	27.6 ± 11.8^a^	30.2 ± 32.2^a^	30.7 ± 24.6^a^	29.8 ± 14.8^a^	29.3 ± 22.2	3 (0.46)
Height (cm)	110 ± 64.6^a^	112 ± 59.2^a^	123 ± 41.4^2^^,b^	116 ± 52.8^a^	114 ± 50.9	4 (0.22)
BMI class^1^ (%)						
Thin	14 (8–42)	9 (3–16)	11 (3–18)	6 (−0.7 to 12)	11 (8–11)	4 (0.88)
Normal	81 (74–88)	83 (75–92)	80 (71–89)	85 (75–95)	82 (78–86)	
Overweight	5 (0.7–8)	5 (0.2–10)	8 (2–15)	6 (−0.8 to 12)	6 (4–8)	
Obese	0.4 (−1.3 to 2)	2 (−1.5 to 6)	1 (−1.7 to 4)	3 (−1.7 to 8)	1 (0.3–3)	
Mother/adult females						
Education (%)						
None	13 (8–18)^a^	28 (42–35)^2^^,b^	8 (4–10)^a^	12 (6–18)^a^	16 (9–19)	18 (<0.001)^3^
Primary	53 (48–58)	56 (50–61)	47 (40–54)	52 (46–59)	52 (47–57)	
Secondary	29 (21–25)	16 (13–23)	37 (29–45)^2^	31 (20–38)	28 (22–32)	
Tertiary	4 (2–7)	1 (0.2–2)	8 (3–9)^2^	5 (2–8)	4 (2–6)	
Occupation (%)						
Student/unemployed	43 (34–52)^a^	57 (46–68)^2^^,a^	32 (20–43)^b^	40 (27–54)^a^	44 (38–48)	9 (0.160)
Informal	35 (27–44)	31 (42–40)	45 (34–56)^2^	35 (20–48)	36 (32–40)	
Salary/manager	22 (15–29)	12 (5–42)	22 (10–32)	24 (10–25)	20 (16–22)	
Marital status (%)						
Single	46 (37–55)	50 (39–61)	48 (37–59)	41 (28–54)	47 (41–51)	12 (0.200)
Partnership	31 (21–38)	37 (27–48)	37 (26–48)	49 (36–63)	37 (33–41)	
Married	22 (15–29)	9 (2–15)	13 (5–23)	9 (1–17)	14 (14–18)	
Divorced	—	—	—	—	—	
Widow	1 (−0.9 to 3)	4 (−0.2 to 8)	2 (−1.1 to 5)	0.4 (−1.3 to 2)	2 (0.6–3)	
Household						
Housing type (%)						
Shack	52 (43–61)^2^^,a^	59 (48–70)^a^	34 (21–45)^b^	44 (31–57)_a,b_	48 (44–53)	11 (0.100)
Apartment	31 (21–38)	32 (23–43)	47 (35–58)^2^	34 (20–47)	35 (31–39)	
Villa	17 (13–22)^2^	9 (2–16)	19 (14–28)	22 (14–33)	16 (10–42)	
Household type (%)						
Other	10 (5–15)	15 (7–20)	7 (1–9)	10 (2–17)	10 (7–10)	9 (0.150)
Monogamy	86 (80–92)	75 (66–85)	92 (86–98)	88 (80–97)	85 (82–88)	
Polygamy	4 (0.9–8)	10 (4–16)^2^	1 (−1.4 to 4)	2 (−2 to 6)	5 (3–7)	
Expenditure (%)						
<2000Fr	20 (10–27)^a^	32 (20–41)^a^	34 (22–45)^a^	20 (12–31)^a^	26 (20–30)	7 (0.280)
2000–4000Fr	58 (50–67)	48 (37–59)	51 (39–62)	61 (48–74)	54 (50–59)	
>4000Fr	21 (11–29)	20 (14–29)	15 (7–22)	19 (12–29)	19 (16–21)	
Number of dependent children	4.2 ± 3^a^	4.4 ± 2.9^a,b^	3.9 ± 2.3^b^	3.9 ± 2.7^b^	4.1 ± 2.4	3 (0.38)
Number of people living in household	7.5 ± 4.4^a^	7.8 ± 4.4^a^	7 ± 3.6^b^	6.9 ± 3.6^b^	7.4 ± 3.6	3 (0.43)

Data are presented as mean ± SD or percentage (95% confidence interval).

Abbreviation: FR, West African Franc, conversion rate in 2021 was 0.0015 euro.

^a,b,c^ Indicate differences between any 2 dietary patterns by pairwise comparison, with similar superscripts indicating no difference.

1 For children *z*-scores cutoffs for BMI/age <−2 SD (thinness), between −2 SD and +1 SD (normal weight), BMI-for-age >+1 SD (overweight), BMI-for-age >+2 SD (obesity), and height-for-age < −2 SD (stunting). For adults, nutritional status was assessed using the BMI (kg/m^2^) cutoffs.

2 Indicates variables that contributed most to the characterization of each dietary pattern.

3 *p*-value <0.05 indicate differences between any two dietary patterns by pairwise comparison.

**TABLE 4:** General characteristics of the urban Ivorian female adult (15–49-y old) population by dietary pattern

	“Dairy and sweet”	“Fish, vegetable, and oil”	“Poultry, red meat, and fruit”	“Low energy intake”	Total	Wald (*P* value)
*n* (%)	104 (22)	139 (32)	112 (26)	79 (18)	434	
kcal/d ± SD	3170 ± 1920	3219 ± 1834	2727 ± 1176	1242 ± 485^2^	2720 ± 1312	50 (<0.001)^3^
Participants						
Age (y)	28.5 ± 8.9^a,b^	30.6 ± 9.9^a^	27.8 ± 9.3^b^	31.1 ± 10.4^a^	29.4 ± 8.8	9 (0.04)^3^
City (%)						
Abengourou	11 (5–17)^a^	10 (4–15)^a^	3 (−1 to 6)^b^	15 (6–21)^c^	9 (6–9)	36 (<0.001)^3^
Abidjan (Youpougon)	19 (14–27)	26 (18–34)	54^2^ (44–64)	41 (29–53)	34 (30–38)	
Bouke	35^2^ (24–44)	27^2^ (19–35)	23 (15–32)	38^2^ (26–50)	30 (26–34)	
Daloa	36 (26–45)^2^	37 (28–46)^2^	20 (9–29)	6 (−1.1 to 13)	27 (21–31)	
Ethnicity (%)						
Akan	30 (23–39)^a^	34 (26–43)^a^	63^2^ (53–73)^b^	65^2^ (53–77)^b^	46 (41–51)	40 (0.00)^3^
Gour	8 (3–11)	11^2^ (6–17)	1 (−1.6 to 4)	5 (−0.8 to 11)	7 (5–12)	
Krou	11 (4–17)	19^2^ (9–26)	8 (2–10)	13 (5–23)	13 (13–16)	
Mande du Nord	23^2^ (15–32)	17 (13–22)	18^2^ (13–26)	4 (−1.6 to 9)	16 (10–42)	
Mande du Sud	8 (3–11)	9^2^ (4–11)	4 (−0.2 to 8)	5 (−0.5 to 10)	7 (4–12)	
Non-Ivorian	11^2^ (5–17)	5 (1–13)	3 (−1.1 to 6)	8 (0.8–15)	6 (4–12)	
Other	8^2^ (3–11)	4 (0.8–8)	4 (−0.3 to 7)	0.7 (−2.2 to 4)	4 (2–6)	
Weight (kg)	8^2^ (3–11)	4 (0.8–8)	4 (−0.3 to 7)	0.7 (−2.2 to 4)	4 (2–6)	1 (0.86)
Height (cm)	128 ± 62.8^a^	128 ± 83.2^a^	155^2^ ± 49.9^b^	160 ± 6^2^^,b^	141 ± 56	59 (<0.001)^3^
BMI class^1^ (%)						
Thin	4^2^ (0–8)^a^	2 (−0.5 to 5)^a^	2 (−1.2 to 5)^a^	1 (−1.9 to 5)^a^	2 (0.9–4)	12 (0.24)
Normal	41 (31–51)	48^2^ (39–58)	53^2^ (43–64)	39 (26–51)	46 (40–51)	
Overweight	40 (30–50)	26 (18–34)	30 (42–38)	45 (32–57)^2^	33 (29–37)	
Obese	15 (8–21)	23^2^ (16–31)	15 (7–21)	15 (6–24)	18 (11–20)	
Mother/adult females						
Education (%)						
None	23 (16–30)^a^	33 (24–40)^2^^,a^	15 (12–42)^2^^,b^	28 (19–36)^a^	25 (23–29)	15 (0.00)^3^
Primary	24 (19–28)	26 (23–30)	20 (16–24)	25 (23–30)	24 (42–28)	
Secondary	39 (33–45)	32 (26–37)	44 (37–50)	36 (28–43)	38 (33–41)	
Tertiary	14 (12–42)	9 (5–10)	21 (15–28)^2^	11 (6–16)	14 (14–17)	
Occupation (%)						
Student/						
Unemployed	39 (29–49)^a^	28 (42–36)^a^	45 (35–55)^2^^,b^	30 (19–40)^a^	35 (31–39)	37 (<0.001)^3^
Informal	43 (33–53)	62 (53–71)^2^	41 (32–51)	48 (36–61)	50 (45–54)	
Salary/manager	18 (13–26)	10 (4–16)	14 (7–23)	22 (9–32)	15 (9–18)	
Marital status (%)						
Single	48 (38–58)	43 (34–52)	62^2^ (52–72)	37 (24–49)	48 (43–53)	6 (0.43)
Partnership	33 (22–43)	36 (28–45)	28^2^ (19–36)	31 (42–41)	32 (28–36)	
Married	18 (13–24)	18 (14–24)	6^2^ (1–9)	29 (18–39)	17 (10–42)	
Divorced	0.8 (−1.1 to 3)	0.8 (−0.8 to 2)	3 (−0.5 to 6)	0 (0–0)	1 (0.2–2)	
Widow	0.9 (−1.1 to 3)	2 (−0.6 to 5)	0.7 (−1.4 to 3)	3 (−1.5 to 7)	2 (0.4–3)	
Household						
Housing type (%)						
Shack	48 (38–58)^a,b^	50 (41–59)^2^^,a^	42 (32–52)^a,b^	34 (20–46)^b^	44 (39–49)	113 (<0.001)^3^
Apartment	32 (21–40)	29 (23–36)	40 (30–50)^2^	45 (32–57)^2^	35 (31–39)	
Villa	20 (9–28)	21 (10–28)	18 (13–26)	22 (9–32)	20 (16–22)	
Household type (%)						
Other	22 (10–30)	21 (11–29)	26 (17–35)	19 (12–29)	22 (18–26)	4 (0.67)
Monogamy	70 (61–79)	68 (60–77)	74 (65–83)	78 (67–88)	72 (68–76)	
Polygamy	9 (3–11)	11 (5–16)	0 (0–0)^2^	3 (−1.7 to 8)	6 (4–8)	
Expenditure (%)						
<2000Fr	25 (16–34)^a^	17 (13–21)^a^	20 (14–28)^a^	24 (10–34)^a^	21 (17–24)	7 (0.28)
2000–4000Fr	61 (51–71)	66 (57–74)	60 (50–70)	55 (43–68)	61 (57–66)	
>4000Fr	14 (7–23)	18 (14–24)	20 (9–28)	21 (14–31)	18 (11–20)	
Number of dependent children	2.6 ± 2.7^a^	3.1 ± 3.5^a^	1.9 ± 2.1^2^^,b^	3.1 ± 3.1^a^	2.7 ± 2.7	16 (0.00)^3^
Number of people living in household	6.6 ± 3.9^a^	7.1 ± 5.3^a^	6.2 ± 3.9^a^	6.5 ± 5.1^a^	6.6 ± 4.3	2 (0.52)

Data are presented as mean ± SD or percentage (95% confidence interval).

Abbreviation: FR, West African Franc, conversion rate in 2021 was 0.0015.

^a,b,^^c^Indicate differences between any 2 dietary patterns by pairwise comparison, with similar superscripts indicating no difference.

1 For children *z*-scores cutoffs for BMI/age <−2 SD (thinness), between −2 SD and +1 SD (normal weight), BMI-for-age >+1 SD (overweight), BMI-for-age >+2 SD (obesity), and height-for-age <−2 SD (stunting). For adults, nutritional status was assessed using the BMI (kg/m^2^) cutoffs.

2 Indicates variables that contributed most to the characterization of each dietary pattern.

3 *p*-value <0.05 indicate differences between any two dietary patterns by pairwise comparison.

In both groups, participants in the “Fish, vegetables, and oil” and “Dairy and sweet” patterns were more often living in Bouaké and Daloa and more likely to live in a “common commune or shack.” The “Fish, vegetables, and oil” pattern was associated with lower education of both the children’s primary caregivers and adult females. Specifically, 28% of children’s primary caregivers in this pattern had no education, compared with 8% to 13% in the other 3 patterns. Similarly, 33% of adult females in this pattern had no education, compared with 15% to 28% in the other patterns. Children’s primary caregiver in this pattern was also more frequently engaged in informal work (62% vs. 41%–48% in the other patterns).

The “Poultry and fruit” pattern in children and “Poultry, red meat, and fruit” pattern in adult females showed several contrasts with the other patterns. Participants were predominantly from Abidjan (53% of children’s primary caregivers and 54% of adult females) and had higher education levels. Specifically, 37% of children’s primary caregivers and 44% of adult females in this pattern had completed secondary education, compared with 16% to 31% and 32% to 39%, respectively, in the other patterns. Among children, their primary caregivers were most often engaged in informal work (45% vs. 31%–35% in other patterns), whereas adult females in this pattern were younger (mean 27.8 ± 9.3), included a higher proportion of unemployed or students (57% vs. 32%–43%), and had fewer dependent children (mean 1.9 ±2.1).

Among adult females, the “Low energy intake” pattern was more common in Abidjan and Bouaké and was associated with a higher proportion living in an apartment (45% vs. 29%–40% in the other patterns). Among children, the “Refined, red meat, and vegetable” pattern was distributed across all the 4 cities and showed no significant sociodemographic differences compared with the other patterns.

### Associations between dietary patterns and nutritional status

No significant differences in nutritional status were observed across dietary patterns in either children (Table 3) or adult females (Table 4). The only exception was mean height among adult females, which was higher in the “Low energy intake” pattern (160 ± 6 cm) than the other 3 patterns (128–155 cm).

## Discussion

This study identified 4 dietary patterns in both children and adult females in urban Côte d’Ivoire. Two patterns, the “Fish, vegetable, and oil” and the “Dairy and sweet,” were common to both population groups, suggesting shared dietary behaviors. These patterns, together with the “Refined, red meat, and vegetable” for children, were associated with healthier diets in terms of lower risk of micronutrient inadequacies and NCDs (GDQS), higher diet diversity (DDS), and nutrient adequacy, than the “Poultry and fruit” pattern in children and the “Low energy intake” pattern in adult females. Healthier patterns were more common in Bouaké and Daloa (outside of Abidjan) and among participants with lower education, whereas the less healthy were more common in Abidjan, among those with higher socioeconomic indicators.

Our findings align with Popkin’s model of nutrition transition [11], in which cereal-based diets shift toward animal-sourced foods, cheaper (vegetable) oils, added sugars, and energy-dense nutrient-poor processed foods as urbanization increases [[38], [39], [40]] and income rises [41]. The inclusion of both staple and animal-sourced foods across all identified patterns suggests that Ivorian urban diets already reflect this transition, differing from earlier reports in urban populations in Nigeria, Ghana, and Burkina Faso, which described predominantly starchy diets with limited animal products [[42], [43], [44]].

The “Dairy and sweet” pattern identified in our study resembles those previously reported for adult populations in urban Nigeria (“Dairy and sugar”) and Ghana (“Sweet tooth”) [42,43]. However, unlike the Nigerian “Dairy and sugar” pattern, which had the lowest GDQS total (because of higher GDQS−), our “Dairy and sweet” maintained a moderate total GDQS and DDS because of the concurrent consumption of foods considered healthy (higher GDQS+) and unhealthy (higher GDQS−). In all 3 studies, sweets, primarily derived from baked goods, sugar-sweetened beverages, and added sugars in drinks, consistently formed the main unhealthy dietary component [42,43]. These similarities suggest emerging convergence in urban dietary patterns across West Africa as unhealthy and sweetened foods become more available.

Diet quality varied across dietary patterns but remained suboptimal overall. Most participants were classified as being at high risk of nutrient inadequacy and NCDs by the GDQS, and few met the DDS criterion of consuming ≥5 food groups. Across indicators, higher GDQS and DDS values were generally in accordance with greater nutrient adequacy, except in the “Fish, oil, and vegetable” pattern among children, which showed a high GDQS but low DDS and intermediate nutrient adequacy compared with other patterns. This reflects key differences between the 2 supplement indicators. DDS captures the number of food groups consumed, whereas GDQS accounts for both food quality (healthy and unhealthy) and quantity. Diets dominated by a few nutrient-dense foods can therefore score well on GDQS while remaining limited in diversity.

Although overall diet quality was poor in both population groups, the relatively healthier patterns, especially the “Dairy and sweet,” suggest some dietary diversification within the urban settings studied. This observation is consistent with the broader regional evidence that urban food environments in West Africa (Nigeria, Senegal, and Mali) are becoming more diverse, as reported by the Organization for Economic Co-operation and Development/Sahel and West Africa Club based on national household data from 2014–2015 to 2018–2019 [9]. Our results therefore reflect within-urban variation, suggesting that diversification in urban food environments may also extend beyond major metropolitan centers.

Recent household survey and market data from West African cities indicate a continued reliance on traditional staples alongside a gradual increase in packaged and sweetened products [9]. Daily sugar-sweetened beverage intake in our population (194–327 g/d) remains well below levels reported in Latin America (500–800 g/d), consistent with an early phase of the nutrition transition and a remaining window for preventive policy action [38,45].

The higher prevalence of overweight and obesity in adult females than in children observed in our study could not be explained by dietary patterns. Despite differences in energy intake (Supplemental Tables 4 and 5), diet types and quality were broadly similar across children and adult females, consistent with global evidence across most regions [46]. Body weight differences may be more strongly influenced by total energy intake, physical activity, or sociocultural norms rather than diet quality alone. In sub-Saharan Africa, higher body weight is often culturally valued among adult females [47] and urbanization has reduced physical activity. Research should continue to prioritize adult females, focusing on biological, behavioral, and sociocultural pathways that heighten vulnerability to obesity in changing food environments [7,48].

The “Low energy intake” pattern in adult females warrants careful interpretation, as it showed the lowest mean energy intake (∼1242 kcal/d), poorest diet quality, and the highest prevalence of overweight/obesity (∼60%), suggesting underreporting, a common bias in dietary recalls especially among overweight individuals [49]. Adult females in this pattern were also taller on average, implying higher energy needs and reinforcing the likelihood of underestimation. Underreporting likely affected this pattern; if food groups rather than amounts were omitted, pattern validity would be compromised.

Dietary patterns were strongly shaped by urbanicity and socioeconomic context, reflecting an early but heterogeneous stage of the nutrition transition. Healthier patterns were associated with lower education and residence outside of Abidjan, whereas less healthy “Poultry” patterns were more common among higher socioeconomic groups in Abidjan—consistent with observations from other LMICs where higher socioeconomic status is often associated with less healthy diets, unlike high-income countries [11,46,50]. This suggests that urbanity may drive a shift away from traditional or common dietary practices (such as the fish, green leafy vegetables, and oil) even when these are healthier. However, these findings do not fully align with classic descriptions of the nutrition transition, which emphasize increased consumption of energy-dense, ultra-processed foods among wealthier urban groups [11].

This complexity aligns with global evidence showing that traditional sociodemographic indicators such as education and urbanicity do not consistently predict diet quality across contexts [46]. Instead, food environment characteristics, such as food access, affordability, and retail infrastructure, may be more sensitive predictors of dietary patterns in LMICs [51,52]. For example, a World Bank report found through a case study using household survey interviews that many urban Ivorian consumers prioritized retailer’s proximity over price or quality [53].

The main strength of this study is that, to our knowledge, it is the first to characterize dietary patterns among children and adult females in Côte d’Ivoire using a data-driven approach. Including both population groups within the same analytical framework allowed the identification of shared and distinct dietary patterns, providing insight into common household-level behaviors and strengthening the relevance of findings for interventions that target families rather than individuals alone. Dietary patterns were identified using LCA, a probabilistic, model-based approach that accounts for uncertainty in class membership and reduces misclassification. Unlike PCA, which produces continuous factor scores, LCA yields discrete, interpretable dietary patterns particularly suited to skewed dietary intake data common in low-income settings [23,42]. In addition, the use of a bias-adjusted 3-step method allowed for correction of classification error, resulting in more accurate estimations of associations between dietary pattern and other variables.

This study has several limitations. First, data were collected in 4 urban cities of Côte d’Ivoire and may not represent national diets. Second, potential underreporting, particularly among adult females, in whom overweight and obesity are common, as mentioned above, may have affected our results. Third, dietary patterns and diet quality were characterized using a single 24-h recall in the LCA method, which does not capture usual intake. Nutrient adequacy bootstrapping was restricted to 50 iterations because of small subgroup numbers, which may have affected precision.

In summary, 4 dietary patterns were identified in urban Ivorian children and adult females. Patterns rich in fish, vegetables, and dairy were associated with higher diet quality and nutrient adequacy, whereas poultry- and low-intake patterns showed poorer quality. These findings highlight opportunities to strengthen culturally familiar, nutrient-dense dietary behaviors in urban populations.

## Author contributions

The authors’ responsibilities were as follows – KA, AM-B, EFT: designed research; GT, AGK, MG: conducted research; KA: analyzed data; KA, EFT, KM, AM-B: wrote the manuscript; KA: had primary responsibility for final content; and all authors: read and approved the final manuscript.

## Data availability

Data described in the manuscript, code book, and analytic code are available upon request.

## Declaration of Generative AI and AI-assisted technologies in the writing process

During the preparation of this work, the authors used ChatGPT for grammatical checks and sentence rewording. After using this tool, the authors reviewed and edited the content as needed and take full responsibility for the content of the publication.

## Funding

FrieslandCampina funded the study, and KA and IB-O are employed by FrieslandCampina. Additionally, the Ivory Coast Nutrition Society research team received funding from FrieslandCampina to conduct this research.

## Conflict of interest

The authors report no conflicts of interest.
