# Supplementary material for: Dietary Patterns and Nutritional Status of Urban School-Children and Adult Females of Reproductive Age in Côte d’Ivoire
Source: Curr Dev Nutr. 2026 Jun 30;10(8):109419. doi: 10.1016/j.cdnut.2026.109419 (PMC13451884; doi:10.1016/j.cdnut.2026.109419)
Supplement: Supplementary file 1 [file mmc1.docx]

# **Dietary patterns and nutritional status of urban school-children and adult females of reproductive age in Côte d’Ivoire. Ayling K- Supplementary material**

**Supplement Table 1.** Profile of each food group^1^ stratified by dietary pattern cluster for children

|  | Cluster1 | Cluster2 | Cluster3 | Cluster4 | Overall | Wald (p-value) |
| --- | --- | --- | --- | --- | --- | --- |
| Cluster Size | 0.36 | 0.25 | 0.23 | 0.16 | 435 |  |
| Citrus fruits |  |  |  |  |  |  |
| Non-consumers | 92 (87–97) | 100 (100–100) | 97 (94–101) | 92 (84–99) | 95 (93–97) | 120 (<0.001) |
| Consumers | 8 (3–13) | 0 (0–0) | 3 (-1–6) | 8 (1–16) | 5 (3–7) |  |
| Deep orange fruits |  |  |  |  |  |  |
| Non-consumers | 100 (100–100) | 89 (83–96) | 98 (95–102) | 93 (87–99) | 96 (94–98) | 180 (<0.001) |
| Consumers | 0 (0–0) | 11 (4–17) | 2 (-1.8–5) | 7 (0.5–13) | 4 (2–6) |  |
| Other fruits |  |  |  |  |  |  |
| Non-consumers | 89 (84–94) | 100 (100–100) | 83 (75–91) | 97 (92–102) | 92 (89–94) | 209 (<0.001) |
| Consumers | 11 (6–16) | 0 (0–0) | 17 (9–25) | 3 (-2.1–8) | 8 (6–11) |  |
| Dark green leafy vegetables |  |  |  |  |  |  |
| Non-consumers | 95 (91–99) | 73 (64–83) | 100 (100–100) | 92 (84–100) | 90 (88–93) | 250 (<0.001) |
| Consumers | 5 (0.5–9) | 27 (17–36) | 0 (0–0) | 8 (0.2–16) | 10 (7–12) |  |
| Other vegetables |  |  |  |  |  |  |
| Below Median | 52 (43–61) | 33 (23–44) | 78 (68–88) | 56 (43–70) | 54 (49–59) | 23 (<0.001) |
| Above Median | 48 (39–57) | 67 (56–77) | 22 (12–32) | 44 (30–57) | 46 (41–51) |  |
| Legumes |  |  |  |  |  |  |
| Non-consumers | 100 (100–100) | 93 (87–98) | 82 (74–90) | 85 (75–94) | 91 (89–94) | 35 (<0.001) |
| Consumers | 0 (0–0.1) | 7 (2–13) | 18 (10–26) | 15 (6–25) | 9 (6–11) |  |
| Nuts and seeds |  |  |  |  |  |  |
| Non-consumers | 77 (69–84) | 63 (52–74) | 96 (90–102) | 74 (62–86) | 77 (73–81) | 11 (0.012) |
| Consumers | 23 (16–31) | 37 (26–48) | 4 (-2–10) | 26 (14–38) | 23 (19–27) |  |
| Liquid oils |  |  |  |  |  |  |
| Non-consumers | 5 (0.1–11) | 18 (9–27) | 38 (27–49) | 48 (35–62) | 23 (19–27) | 31 (<0.001) |
| Below | 44 (35–53) | 46 (35–57) | 35 (25–46) | 26 (13–38) | 39 (35–44) |  |
| Above | 51 (42–60) | 36 (26–47) | 27 (16–37) | 26 (14–38) | 38 (33–42) |  |
| Fish and shellfish |  |  |  |  |  |  |
| Non-consumers | 7 (2–13) | 0.1 (0–0.1) | 45 (34–56) | 44 (31–57) | 20 (17–24) | 547 (<0.001) |
| below | 39 (31–48) | 55 (44–65) | 31 (20–41) | 38 (25–51) | 41 (36–45) |  |
| above | 53 (45–62) | 45 (35–56) | 24 (14–34) | 18 (6–29) | 39 (34–43) |  |
| Poultry and game meat |  |  |  |  |  |  |
| Non-consumers | 88 (82–94) | 94 (88–100) | 78 (69–87) | 99 (94–103) | 89 (86–92) | 10 (0.018) |
| Consumers | 12 (6–18) | 6 (0.1–12) | 22 (13–31) | 1 (-3–6) | 11 (8–14) |  |
| Eggs |  |  |  |  |  |  |
| Non-consumers | 77 (69–84) | 100 (100–100) | 52 (41–63) | 75 (64–86) | 76 (73–80) | 222 (<0.001) |
| Consumers | 23 (16–31) | 0.1 (0–0.2) | 48 (37–59) | 25 (14–36) | 24 (20–27) |  |
| High fat dairy |  |  |  |  |  |  |
| Non-consumers | 27 (18–35) | 94 (88–101) | 99 (95–102) | 80 (69–91) | 69 (65–72) | 60 (<0.001) |
| Consumers | 73 (65–82) | 6 (-1–12) | 1 (-2.1–5) | 20 (9–31) | 31 (28–35) |  |
| Red meat |  |  |  |  |  |  |
| Non-consumers | 39 (31–48) | 51 (40–62) | 49 (38–60) | 45 (32–58) | 45 (41–50) | 25 (<0.001) |
| Below | 51 (42–59) | 44 (33–55) | 25 (15–36) | 21 (9–32) | 38 (34–43) |  |
| Above | 10 (4–16) | 5 (-1–12) | 26 (16–35) | 35 (22–47) | 17 (13–20) |  |
| Refined grains and baked goods |  |  |  |  |  |  |
| Below Median | 58 (49–67) | 49 (38–60) | 99 (97–101) | 0.2 (0.1–0.3) | 56 (52–60) | 780 (<0.001) |
| Above Median | 42 (33–51) | 51 (40–62) | 0.9 (-0.9–3) | 100 (100–100) | 44 (40–48) |  |
| Sweets and ice cream |  |  |  |  |  |  |
| Non-consumers | 0.1 (0.1–0.1) | 84 (76–92) | 72 (61–82) | 30 (18–43) | 42 (39–46) | 1393 (<0.001) |
| Below | 52 (44–60) | 16 (8–24) | 17 (8–26) | 26 (14–38) | 31 (27–35) |  |
| Above | 48 (40–56) | 0.3 (-0.1–0.7) | 11 (4–19) | 43 (30–57) | 27 (23–31) |  |
| Sugar sweetened beverages |  |  |  |  |  |  |
| Non-consumers | 94 (90–98) | 100 (100–100) | 90 (83–96) | 96 (90–102) | 95 (93–97) | 11 (0.014) |
| Consumers | 6 (2–10) | 0 (-0.1–0.2) | 10 (4–17) | 4 (-1.8–10) | 5 (3–7) |  |
| Juice |  |  |  |  |  |  |
| Non-consumers | 50 (41–59) | 100 (99–100) | 57 (46–68) | 69 (56–81) | 67 (63–71) | 64 (<0.001) |
| Consumers | 50 (41–59) | 0.4 (-0.1–0.8) | 43 (32–54) | 31 (19–44) | 33 (29–37) |  |
| White roots and tubers |  |  |  |  |  |  |
| Non-consumers | 7 (2–12) | 3 (-2.3–8) | 12 (5–19) | 69 (58–81) | 17 (14–20) | 508 (<0.001) |
| Below | 47 (38–55) | 62 (51–73) | 65 (54–76) | 31 (19–42) | 52 (47–57) |  |
| Above | 46 (37–55) | 35 (25–46) | 23 (13–33) | 0.1 (0.1–0.2) | 31 (26–35) |  |
| Cruciferous vegetables |  |  |  |  |  |  |
| Non-consumers | 98 (96–100) | 99 (97–101) | 100 (100–100) | 100 (100–100) | 99 (98–100) | 27 (<0.001) |
| Consumers | 2 (-0.3–4) | 1 (-1–3) | 0 (0–0) | 0 (0–0) | 0.9 (0–2) |  |
| Deep orange vegetables |  |  |  |  |  |  |
| Non-consumers | 98 (96–100) | 100 (100–100) | 99 (97–101) | 100 (100–100) | 99 (98–100) | 24 (<0.001) |
| Consumers | 2 (-0.4–4) | 0 (0–0) | 1 (-1.4–3) | 0 (0–0) | 0.9 (0–2) |  |
| Deep orange tubers |  |  |  |  |  |  |
| Non-consumers | 100 | 100 | 100 | 100 | 100 |  |
| Whole grains |  |  |  |  |  |  |
| Non-consumers | 97 (94–100) | 99 (98–101) | 100 (98–101) | 98 (95–101) | 98 (97–99) | 5 (0.180) |
| Consumers | 3 (0.5–6) | 0.8 (-0.6–2) | 0.5 (-0.9–2) | 2 (-1.4–5) | 2 (0.6–3) |  |
| Low fat dairy |  |  |  |  |  |  |
| Non-consumers | 100 | 100 | 100 | 100 | 100 |  |
| Processed meat |  |  |  |  |  |  |
| Non-consumers | 99 (98–101) | 100 (99–100) | 100 (100–100) | 100 (100–100) | 100 (99–100) | 24 (<0.001) |
| Consumers | 0.5 (-0.5–2) | 0.2 (-0.2–0.6) | 0 (0–0) | 0 (0–0) | 0.2 (-0.2–0.7) |  |
| Purchased deep fried foods |  |  |  |  |  |  |
| Non-consumers | 100 | 100 | 100 | 100 | 100 |  |

^1^ Food items were grouped into the food groups of the Global Diet Quality Score

All values reported as % (95% CI) unless specified

**Supplement Table 2.** Profile of each food group^1^ stratified by dietary pattern cluster for adult females

|  | Cluster1 | Cluster2 | Cluster3 | Cluster 4 | Overall | Wald (p-value) |
| --- | --- | --- | --- | --- | --- | --- |
| Cluster Size | 0.3 | 0.26 | 0.24 | 0.18 | 434 |  |
| Citrus fruits |  |  |  |  |  |  |
| Non-consumers | 98 (96–101) | 94 (90–99) | 97 (93–100) | 100 (100–100) | 97 (96–99) | 50 (<0.001) |
| Consumers | 2 (-0.8–4) | 6 (1–10) | 3 (-0.2–7) | 0 (0–0) | 3 (1–4) |  |
| Deep orange fruits |  |  |  |  |  |  |
| Non-consumers | 96 (92–99) | 96 (92–100) | 96 (92–100) | 100 (100–100) | 97 (95–98) | 4 (0.2800) |
| Consumers | 4 (0.8–8) | 4 (0.1–8) | 4 (-0.1–8) | 0 (-0.2–0.3) | 3 (2–5) |  |
| Other fruits |  |  |  |  |  |  |
| Non-consumers | 95 (92–99) | 96 (92–100) | 90 (85–96) | 96 (91–101) | 94 (92–97) | 3 (0.3200) |
| Consumers | 5 (0.8–8) | 4 (0.2–8) | 10 (4–15) | 4 (-1–9) | 6 (3–8) |  |
| Dark green leafy vegetables |  |  |  |  |  |  |
| Non-consumers | 84 (78–91) | 94 (88–99) | 84 (77–91) | 86 (78–95) | 87 (84–90) | 4 (0.2500) |
| Consumers | 16 (9–22) | 6 (1–12) | 16 (9–23) | 14 (5–22) | 13 (10–16) |  |
| Other vegetables |  |  |  |  |  |  |
| Non-consumers | 1 (-2.1–4) | 12 (6–19) | 6 (1–11) | 29 (18–40) | 10 (8–13) | 46 (<0.001) |
| Consumers | 24 (16–32) | 55 (44–65) | 44 (34–54) | 70 (59–80) | 45 (40–49) |  |
| Legumes |  |  |  |  |  |  |
| Non-consumers | 97 (94–100) | 95 (91–99) | 94 (90–99) | 100 (100–100) | 96 (95–98) | 65 (<0.001) |
| Consumers | 3 (0.1–6) | 5 (0.7–9) | 6 (1–10) | 0 (0–0) | 4 (2–5) |  |
| Nuts and seeds |  |  |  |  |  |  |
| Non-consumers | 74 (67–82) | 85 (77–92) | 76 (68–85) | 92 (84–99) | 81 (77–84) | 8 (0.0500) |
| Consumers | 26 (18–33) | 15 (8–23) | 24 (15–32) | 8 (1–16) | 19 (16–23) |  |
| Liquid oils |  |  |  |  |  |  |
| non-consumers | 16 (9–23) | 13 (6–20) | 22 (13–30) | 38 (26–50) | 20 (17–24) | 30 (<0.001) |
| below | 31 (22–39) | 55 (44–65) | 33 (24–43) | 44 (32–56) | 40 (35–44) |  |
| above | 53 (44–62) | 32 (23–42) | 45 (35–55) | 18 (8–29) | 40 (35–44) |  |
| Fish and shellfish |  |  |  |  |  |  |
| Non-consumers | 6 (1–11) | 18 (10–26) | 18 (10–26) | 49 (38–60) | 20 (16–23) | 278 (<0.001) |
| Below | 33 (24–41) | 48 (38–58) | 34 (25–44) | 51 (39–62) | 40 (36–45) |  |
| Above | 61 (52–70) | 34 (25–44) | 48 (38–58) | 0.2 (0.1–0.4) | 40 (36–44) |  |
| Poultry and game meat |  |  |  |  |  |  |
| Non-consumers | 90 (85–96) | 76 (67–84) | 86 (79–93) | 100 (98–101) | 87 (84–90) | 12 (0.0086) |
| Consumers | 10 (4–15) | 24 (16–33) | 14 (7–21) | 0.3 (-0.9–2) | 13 (10–16) |  |
| Eggs |  |  |  |  |  |  |
| Non-consumers | 72 (64–80) | 81 (73–89) | 56 (46–66) | 98 (93–103) | 75 (71–79) | 19 (<0.001) |
| Consumers | 28 (20–36) | 19 (11–27) | 44 (34–54) | 2 (-3.1–7) | 25 (21–29) |  |
| High fat dairy |  |  |  |  |  |  |
| Non-consumers | 88 (82–94) | 93 (87–99) | 25 (16–34) | 89 (80–97) | 74 (71–78) | 105 (<0.001) |
| Consumers | 12 (6–18) | 7 (1–13) | 75 (66–84) | 11 (3–20) | 26 (22–29) |  |
| Red meat |  |  |  |  |  |  |
| Non-consumers | 50 (41–59) | 50 (39–60) | 46 (36–56) | 30 (18–41) | 45 (41–50) | 18 (0.0053) |
| Below | 27 (18–35) | 19 (10–27) | 33 (23–42) | 51 (39–63) | 30 (26–35) |  |
| Above | 24 (16–31) | 32 (22–41) | 21 (13–30) | 19 (9–29) | 24 (20–28) |  |
| Refined grains and baked goods |  |  |  |  |  |  |
| Non-consumers | 0 (0–0) | 36 (27–44) | 0 (0–0) | 5 (0–10) | 10 (8–13) | 2165 (<0.001) |
| Below | 17 (10–23) | 64 (56–73) | 35 (26–45) | 76 (65–87) | 44 (40–49) |  |
| Above | 83 (77–90) | 0.1 (0.1–0.2) | 65 (55–74) | 18 (8–29) | 45 (42–49) |  |
| Sweets and ice cream |  |  |  |  |  |  |
| Non-consumers | 100 (100–100) | 78 (70–87) | 0.2 (0.1–0.2) | 91 (83–98) | 69 (66–71) | 3033 (<0.001) |
| Consumers | 0.1 (0–0.1) | 22 (13–30) | 100 (100–100) | 9 (2–17) | 31 (29–34) |  |
| Sugar sweetened beverages |  |  |  |  |  |  |
| Non-consumers | 94 (90–98) | 86 (79–93) | 96 (92–100) | 88 (80–96) | 91 (89–94) | 6 (0.1200) |
| Consumers | 6 (2–10) | 14 (7–21) | 4 (-0.3–8) | 12 (4–20) | 9 (6–11) |  |
| Juice |  |  |  |  |  |  |
| Non-consumers | 85 (79–92) | 79 (71–87) | 78 (70–86) | 88 (80–96) | 82 (79–86) | 4 (0.2700) |
| Consumers | 15 (8–21) | 21 (13–29) | 22 (14–30) | 12 (4–20) | 18 (14–21) |  |
| White roots and tubers |  |  |  |  |  |  |
| Non-consumers | 20 (12–27) | 0.1 (0–0.1) | 33 (24–42) | 58 (47–69) | 25 (21–28) | 1511 (<0.001) |
| below | 52 (43–61) | 25 (17–34) | 28 (19–37) | 42 (31–53) | 38 (33–42) |  |
| above | 28 (20–36) | 74 (66–83) | 39 (29–49) | 0.1 (0.1–0.2) | 38 (34–42) |  |
| Cruciferous vegetables |  |  |  |  |  |  |
| Non-consumers | 99 (97–101) | 100 (100–100) | 99 (97–101) | 99 (96–102) | 99 (98–100) | 2 (0.4800) |
| Consumers | 1 (-0.7–3) | 0 (-0.1–0.1) | 1 (-1–3) | 1 (-1.6–4) | 0.9 (0–2) |  |
| Deep orange vegetables |  |  |  |  |  |  |
| Non-consumers | 100 (100–100) | 99 (97–101) | 100 (100–100) | 99 (96–101) | 100 (99–100) | 17 (<0.001) |
| Consumers | 0 (0–0.1) | 0.8 (-0.9–3) | 0 (0–0) | 1 (-1.3–4) | 0.5 (-0.2–1) |  |
| Deep orange tubers |  |  |  |  |  |  |
| Non-consumers | 100 | 100 | 100 | 100 | 100 |  |
| Whole grains |  |  |  |  |  |  |
| Non-consumers | 100 (100–100) | 98 (96–101) | 100 (100–100) | 100 (100–100) | 100 (99–100) | 33 (<0.001) |
| Consumers | 0 (0–0) | 2 (-0.7–4) | 0 (0–0) | 0 (0–0) | 0.5 (-0.2–1) |  |
| Low fat dairy |  |  |  |  |  |  |
| Non-consumers | 100 | 100 | 100 | 100 | 100 |  |
| Processed meat |  |  |  |  |  |  |
| Non-consumers | 100 (100–100) | 99 (97–101) | 99 (97–101) | 100 (100–100) | 100 (99–100) | 18 (<0.001) |
| Consumers | 0 (0–0) | 0.9 (-0.9–3) | 0.9 (-1–3) | 0 (0–0) | 0.5 (-0.2–1) |  |
| Purchased deep fried foods |  |  |  |  |  |  |
| Non-consumers | 100 | 100 | 100 | 100 | 100 (296–-96) |  |

^1^ The Global Diet Quality Score was used to categorise foods into food group

All values reported as % (95% CI) unless specified

**Supplement Table 3.** Comparison of model fit statistics among LCA models for dietary patterns with an increasing number of clusters.

| Population group | Clusters | LL | BIC | AIC | AIC3 | Npar | L² | df | p-value | Bootstrap p | Max. BVR | VLMR | p-value | -2LL Diff | Bootstrap p | Class.Err. | Entropy R² |
| --- | --- | --- | --- | --- | --- | --- | --- | --- | --- | --- | --- | --- | --- | --- | --- | --- | --- |
| Children | 1-Cluster | -4681 | 9502 | 9408 | 9431 | 23 | 4096 | 412 | 1.2e-597 | 0 | 30 |  |  |  |  | 0 | 1 |
|  | 2-Cluster | -4625 | 9536 | 9345 | 9392 | 47 | 3985 | 388 | 5.7e-588 | 0 | 11 | 111 | 0.00 | 111 | 0.00 | 0.12 | 0.59 |
|  | 3-Cluster | -4583 | 9597 | 9308 | 9379 | 71 | 3899 | 364 | 1.6e-583 | 0 | 11 | 85 | 0.00 | 85 | 0.00 | 0.15 | 0.67 |
|  | 4-Cluster | -4541 | 9660 | 9273 | 9368 | 95 | 3816 | 340 | 1.3e-579 | 0 | 11 | 83 | 0.00 | 83 | 0.00 | 0.18 | 0.67 |
|  | 5-Cluster | -4511 | 9745 | 9260 | 9379 | 119 | 3756 | 316 | 2.1e-580 | 0 | 7 | 60 | 0.09 | 60 | 0.04 | 0.16 | 0.72 |
|  | 4-Cluster-modified ^1^ | -4431 | 9596 | 9103 | 9224 | 121 | 8767 | 314 | 2.7e-1612 | 0 | 5 |  |  |  |  | 0.16 | 0.71 |
| Adult females | 1-Cluster | -4657 | 9460 | 9362 | 9386 | 24 | 4091 | 410 | 1.0e-597 | 0.30 | 94 |  |  |  |  | 0.0 | 1.0 |
|  | 2-Cluster | -4576 | 9449 | 9249 | 9298 | 49 | 3928 | 385 | 2.1e-578 | 0.23 | 94 | 163 | 0.00 | 163 | 0.00 | 0.12 | 0.59 |
|  | 3-Cluster | -4515 | 9480 | 9178 | 9252 | 74 | 3807 | 360 | 2.0e-567 | 0.19 | 21 | 121 | 0.01 | 121 | 0.00 | 0.14 | 0.66 |
|  | 4-Cluster | -4479 | 9559 | 9155 | 9254 | 99 | 3734 | 335 | 5.5e-566 | 0.12 | 21 | 73 | 0.01 | 73 | 0.00 | 0.17 | 0.67 |
|  | 5-Cluster | -4446 | 9645 | 9140 | 9264 | 124 | 3669 | 310 | 2.6e-566 | 0.09 | 12 | 65 | 0.02 | 65 | 0.02 | 0.15 | 0.74 |
|  | 4-Cluster- modified ^2^ | -4273 | 9202 | 8762 | 8870 | 108 | 8482 | 326 | 5.8e-1544 | 0.15 | 5 |  |  |  |  | 0.08 | 0.79 |

In all models, the 7 food groups with no or low consumption were removed. Dark orange indicates the final selected models.

^1^ Energy (kcal) was added both as co-variate and as a direct effect on food groups.

^2^ Energy (kcal) was added both as co-variate and as a direct effect on food groups; in addition, residual correlation was allowed between food groups with a high BVR (nuts and seeds and other vegetables; red meat and fish).

Abbreviations: LCA, latent class analysis; LL, log-likelihood; BIC, Bayesian Information Criterion; AIC, Akaike Information Criterion; AIC3, adjusted Akaike Information Criterion; Npar, number of parameters; L², likelihood ratio statistic; df, degrees of freedom; p-value, probability value; Boots. trap p, bootstrap likelihood ratio test p-value; Max. BVR, maximum bivariate residual; VLMR, Vuong–Lo–Mendell–Rubin likelihood ratio test; −2LL Diff, difference in −2 log-likelihood; Class. Err, classification error; Entropy R², entropy-based measure of classification quali

**Supplement Table 4.** Mean (± SD) usual nutrient and food group intake according to dietary pattern for school-aged children (N=434)

|  |  | Cluster 1 | Cluster 2 | Cluster 3 | Cluster 4 | Total |
| --- | --- | --- | --- | --- | --- | --- |
|  | Proportion/n | 0.36 (156) | 0.25 (108) | 0.23 (100) | 0.16 (71) | 435 |
|  | Energy, kcal | 2325 ± 922 | 2156 ± 965 | 1615 ± 808 | 2410 ± 1087 | 2134 ± 786 |
| Usual Nutrient intake | Calcium, mg | 485 ± 392 | 376 ± 605 | 207 ± 134 | 343 ± 263 | 371 ± 344 |
|  | Iron, mg | 12.6 ± 5.8 | 14.7 ± 12.4 | 9.8 ± 6.2 | 16.2 ± 12.2 | 13.1 ± 7.4 |
|  | Zinc, mg | 7.2 ± 2.9 | 6.7 ± 3.5 | 5.2 ± 2.6 | 8.4 ± 4.3 | 6.8 ± 2.7 |
|  | Vitamin A, µg RAE^1^ | 673 ± 1058 | 627 ± 1596 | 444 ± 1005 | 441 ± 692 | 571 ± 935 |
|  | Thiamin, mg | 1 ± 0.49 | 1.1 ± 0.65 | 0.74 ± 0.49 | 1 ± 0.76 | 0.97 ± 0.48 |
|  | Riboflavin, mg | 0.98 ± 0.68 | 0.77 ± 0.88 | 0.72 ± 0.92 | 1.1 ± 1.3 | 0.89 ± 0.72 |
|  | VitB6, mg | 1.5 ± 0.91 | 1.3 ± 1.4 | 1.3 ± 1.1 | 1.3 ± 0.77 | 1.4 ± 0.88 |
|  | VitB12, µg | 5.3 ± 5.3 | 5.4 ± 6.5 | 6.1 ± 11.4 | 5.9 ± 12.8 | 5.6 ± 7 |
|  | Vitamin D, µg | 3.7 ± 2.9 | 3.7 ± 4.1 | 2.6 ± 2.5 | 2.6 ± 2.7 | 3.2 ± 2.6 |
|  | Vitamin C, mg | 74.4 ± 78.2 | 93.4 ± 171 | 59 ± 82.1 | 65 ± 68.7 | 74.1 ± 86.9 |
|  | Folate, µg DFE^2^ | 248 ± 151 | 258 ± 214 | 192 ± 160 | 207 ± 164 | 231 ± 139 |
|  | Niacin, mg | 16 ± 10.2 | 18 ± 14.4 | 12.6 ± 11.5 | 16.8 ± 12.9 | 15.8 ± 10.1 |
| Food group intake (mg^1^/day) | Citrus fruits | 10.7 ± 46.9 | 0 ± 0 | 8.9 ± 53.6 | 10 ± 34.4 | 7.6 ± 40.8 |
|  | Cruciferous vegetables | 2.1 ± 17.5 | 0.5 ± 4.9 | 0 ± 0 | 0 ± 0 | 0.9 ± 11.2 |
|  | Dark green leafy vegetables | 3.8 ± 16.9 | 11.7 ± 30.8 | 0 ± 0 | 4.6 ± 21.1 | 5 ± 20.6 |
|  | Deep orange fruits | 0 ± 0 | 18.1 ± 67.4 | 2.7 ± 18.8 | 17.6 ± 72.6 | 7.6 ± 45 |
|  | Deep orange tubers | 0 ± 0 | 0 ± 0 | 0 ± 0 | 0 ± 0 | 0 ± 0 |
|  | Deep orange vegetables | 0.7 ± 6.9 | 0 ± 0 | 0.9 ± 9 | 0 ± 0 | 0.5 ± 6 |
|  | Eggs | 12.1 ± 26 | 2.6 ± 13.7 | 25.9 ± 36.1 | 13.4 ± 28.4 | 13 ± 27.8 |
|  | Fish and shellfish | 67.2 ± 69.7 | 73.9 ± 76.1 | 33.8 ± 44.7 | 34.4 ± 51.5 | 56.7 ± 66.4 |
|  | High fat dairy | 51.6 ± 112.2 | 7 ± 29.7 | 6.7 ± 52.5 | 16.2 ± 34 | 25.7 ± 79.5 |
|  | Juice | 96.9 ± 153.3 | 10.8 ± 44.7 | 89.9 ± 145 | 63.6 ± 130 | 69.4 ± 135 |
|  | Legumes | 0.9 ± 8.1 | 9.2 ± 38.3 | 21.6 ± 57.8 | 39 ± 110.1 | 13.1 ± 55.3 |
|  | Liquid oils | 122 ± 124 | 117 ± 164 | 67.5 ± 88 | 77.3 ± 148 | 102 ± 134 |
|  | Low Fat dairy | 0 ± 0 | 0 ± 0 | 0 ± 0 | 0 ± 0 | 0 ± 0 |
|  | Nuts and seeds | 16.5 ± 39.5 | 31.9 ± 68.4 | 5.2 ± 21 | 52.3 ± 126 | 23.1 ± 66.4 |
|  | Other fruits | 44.5 ± 278 | 0 ± 0 | 42 ± 106 | 12.6 ± 60.2 | 28.4 ± 183 |
|  | Other vegetables | 98.5 ± 121 | 111 ± 120 | 66 ± 79.6 | 115 ± 180 | 96.9 ± 125 |
|  | Poultry and game meat | 17.1 ± 53.6 | 11.6 ± 47.9 | 26.5 ± 77.2 | 8.2 ± 41.9 | 16.5 ± 57 |
|  | Processed meat | 0.1 ± 0.7 | 0 ± 0 | 0 ± 0 | 0 ± 0 | 0 ± 0.4 |
|  | Purchased deep fried foods | 0 ± 0 | 0 ± 0 | 0 ± 0 | 0 ± 0 | 0 ± 0 |
|  | Red meat | 21.3 ± 28.7 | 16.8 ± 28.3 | 29.3 ± 46.2 | 34.2 ± 56.7 | 23.9 ± 38.5 |
|  | Refined grains & baked goods | 486 ± 311 | 495 ± 301 | 267 ± 167 | 928 ± 360 | 507 ± 352 |
|  | Sugar sweetened beverages | 16.2 ± 86.5 | 2.8 ± 29.1 | 17.2 ± 60.4 | 9.8 ± 52.3 | 12.2 ± 65.9 |
|  | Sweets and ice cream | 34.6 ± 59.5 | 1.2 ± 3.8 | 12.4 ± 40.6 | 25.2 ± 48.4 | 20.3 ± 47.6 |
|  | White roots and tubers | 359 ± 272 | 342 ± 281 | 276 ± 232 | 74.7 ± 124 | 295 ± 267 |
|  | Whole grains | 0.6 ± 3.8 | 0 ± 0 | 0.1 ± 1 | 0.4 ± 2.7 | 0.3 ± 2.6 |

^1^ mg/day is raw mean intake

Abbreviations: RAE, Retinol Activity Equivalent; DFE, Dietary Folate Equivalent, SD; standard deviation

**Supplement Table 5.** Mean (±SD) usual nutrient intake according to dietary pattern for adult females (N=434)

|  |  | Cluster 1 | Cluster 2 | Cluster 3 | Cluster 4 | Total |
| --- | --- | --- | --- | --- | --- | --- |
|  | Proportion/n | 139 (32) | 112 (26) | 104 924) | 79 (18) | 434 |
|  | Energy, kcal | 3219 ± 1834 | 2727 ± 1176 | 3170 ± 1920 | 1242 ± 485 | 2720 ± 1312 |
| Usual Nutrient intake | Calcium, mg | 450 ± 292 | 377 ± 205 | 744 ± 449 | 182 ± 130 | 453 ± 286 |
|  | Iron, mg | 21 ± 13.6 | 16.2 ± 7.1 | 19.6 ± 11.9 | 7 ± 3.2 | 16.9 ± 9.5 |
|  | Zinc, mg | 11.2 ± 7.1 | 8.6 ± 5 | 11.6 ± 8.8 | 4.9 ± 2.3 | 9.5 ± 5.4 |
|  | Vitamin A, µg RAE^1^ | 1486 ± 1685 | 185 ± 147 | 823 ± 826 | 355 ± 577 | 784 ± 1053 |
|  | Thiamin, mg | 1.3 ± 1.1 | 1.5 ± 1.3 | 1.3 ± 0.98 | 0.46 ± 0.26 | 1.2 ± 0.68 |
|  | Riboflavin, mg | 1.2 ± 1.7 | 0.84 ± 0.36 | 1.7 ± 1.5 | 0.39 ± 0.2 | 1.1 ± 0.79 |
|  | VitB6, mg | 2 ± 1.3 | 1.9 ± 1.3 | 2.3 ± 1.3 | 0.75 ± 0.45 | 1.8 ± 1 |
|  | VitB12, µg | 6.8 ± 5.2 | 14.6 ± 13.7 | 5.8 ± 4.4 | 1.9 ± 1.4 | 7.6 ± 7.9 |
|  | Vitamin D, µg | 7.3 ± 6.9 | 3.9 ± 3.3 | 5.4 ± 4 | 1.4 ± 1.2 | 4.9 ± 4.5 |
|  | Vitamin C, mg | 81.6 ± 72.8 | 182 ± 141 | 92.3 ± 114 | 30.3 ± 46.8 | 101 ± 92.1 |
|  | Folate, µg DFE^2^ | 270 ± 153 | 463 ± 298 | 286 ± 136 | 98.1 ± 67.7 | 292 ± 187 |
|  | Niacin, mg | 27.2 ± 19.4 | 22.6 ± 19.2 | 24.2 ± 19.1 | 9.5 ± 4.2 | 22.1 ± 13.8 |
| Food group intake (mg^1^/day) | Citrus fruits | 4 ± 28.8 | 8.1 ± 45.8 | 6.6 ± 36.9 | 0 ± 0 | 5 ± 33.9 |
|  | Cruciferous vegetables | 0.7 ± 6 | 0 ± 0 | 0.9 ± 9.7 | 1.3 ± 11.5 | 0.7 ± 7.6 |
|  | Dark green leafy vegetables | 10.5 ± 31.8 | 4.7 ± 23.4 | 8.3 ± 21.6 | 8.8 ± 28 | 8.2 ± 26.9 |
|  | Deep orange fruits | 16.6 ± 99.2 | 11.2 ± 68.1 | 8.7 ± 50.7 | 0 ± 0 | 10.4 ± 70.9 |
|  | Deep orange tubers | 0 ± 0 | 0 ± 0 | 0 ± 0 | 0 ± 0 | 0 ± 0 |
|  | Deep orange vegetables | 0 ± 0 | 1.6 ± 16.4 | 0 ± 0 | 1.1 ± 10 | 0.6 ± 9.4 |
|  | Eggs | 22.3 ± 42.5 | 12.9 ± 34 | 44.3 ± 73.4 | 2.7 ± 11.6 | 21.8 ± 49.3 |
|  | Fish and shellfish | 130 ± 119 | 87 ± 98.1 | 106 ± 123 | 22.8 ± 30.3 | 94.3 ± 110 |
|  | High fat dairy | 11.7 ± 47.1 | 16.2 ± 92.1 | 49.8 ± 69.9 | 4.4 ± 16.8 | 20.9 ± 66.6 |
|  | Juice | 37.5 ± 119 | 54.4 ± 128 | 82.8 ± 198 | 31.7 ± 96.7 | 51.9 ± 141 |
|  | Legumes | 5 ± 34.2 | 6.7 ± 35.1 | 19 ± 108 | 0 ± 0 | 8 ± 59.8 |
|  | Liquid oils | 171 ± 232 | 98.1 ± 118 | 144 ± 210 | 52.4 ± 74.6 | 125 ± 186 |
|  | Low Fat dairy | 0 ± 0 | 0 ± 0 | 0 ± 0 | 0 ± 0 | 0 ± 0 |
|  | Nuts and seeds | 29.6 ± 67.2 | 16.2 ± 55.3 | 35.4 ± 89.3 | 6.9 ± 25.5 | 23.6 ± 66.4 |
|  | Other fruits | 3.6 ± 18.6 | 5.8 ± 31.1 | 22.9 ± 104 | 1.9 ± 9.9 | 8.6 ± 55.4 |
|  | Other vegetables | 174 ± 2010 | 93.7 ± 111 | 1545 ± 185 | 58.5 ± 133 | 128 ± 176 |
|  | Poultry and game meat | 12.4 ± 47.2 | 35.7 ± 89 | 18.4 ± 53.8 | 2.6 ± 23.1 | 18.2 ± 60.8 |
|  | Processed meat | 0 ± 0 | 2.2 ± 23.7 | 4.7 ± 48.7 | 0 ± 0 | 1.7 ± 27 |
|  | Purchased deep fried foods | 0 ± 0 | 0 ± 0 | 0 ± 0 | 0 ± 0 | 0 ± 0 |
|  | Red meat | 37.1 ± 54.6 | 50.9 ± 85 | 31.4 ± 45.5 | 36 ± 36.4 | 39.1 ± 60.1 |
|  | Refined grains & baked goods | 889 ± 503 | 215 ± 203 | 821 ± 567 | 423 ± 302 | 617 ± 519 |
|  | Sugar sweetened beverages | 26.1 ± 103 | 62.7 ± 218 | 14.9 ± 76.4 | 26 ± 94.5 | 32.8 ± 138 |
|  | Sweets and ice cream | 0 ± 0 | 21.7 ± 84.4 | 42.6 ± 80.7 | 4.8 ± 19.7 | 16.8 ± 61.5 |
|  | White roots and tubers | 390± 398 | 667± 340 | 426 ± 498 | 117± 151 | 422 ± 421 |
|  | Whole grains | 0 ± 0 | 0.8 ± 7.4 | 0 ± 0 | 0 ± 0 | 0.2 ± 3.8 |

^1^ mg/day is raw mean intake

Abbreviations: RAE, Retinol Activity Equivalent; DFE, Dietary Folate Equivalent SD; standard deviation
